# Relationship between psychological capital and quality of life among seniors working after retirement: The mediating role of hope of success

**DOI:** 10.1371/journal.pone.0259273

**Published:** 2021-11-09

**Authors:** Krzysztof Jurek, Iwona Niewiadomska

**Affiliations:** 1 Department of Sociology of Culture, Religion and Migration, John Paul II Catholic University of Lublin, Lublin, Poland; 2 Department of Social Psychoprevention, John Paul II Catholic University of Lublin, Lublin, Poland; Tabriz University of Medical Sciences, ISLAMIC REPUBLIC OF IRAN

## Abstract

**Introduction:**

As a result of the increasing average human life expectancy and the related population growth in many countries, research on factors increasing seniors’ quality of life is becoming particularly important. An event critical for seniors’ functioning is retirement. A concept reflecting the dynamics of seniors’ personality associated with the changes taking place in their life situation is psychological capital. This concept was identified as a factor that is constituted by four personality variables changing because of experiences: self-efficacy, optimism, psychological resilience, and hope of success. An interesting issue is the relationship between psychological capital and quality of life in seniors working after retirement.

**Aim:**

The aim of this paper was to analyze the relationship between psychological capital (self-efficacy, dispositional optimism, psychological resilience) and quality of life in seniors working after retirement, as well as the mediating role of hope of success in this relationship.

**Methods:**

A total of 304 seniors—103 women and 201 men—participated in the study. The mean age was 65.24. The Quality-of-Life Scale (CASP-19), the Polish adaptation of Life Orientation Test (LOT-R), the Generalized Self-Efficacy Scale (GSES), the Hope of Success Questionnaire (KNS), and polish adaptation of the Resilience Assessment Scale (SPP-25) were employed in the research. A mediation model was applied to explore the pathway from psychological capital via hope of success to quality of life.

**Results:**

The tested components of psychological capital correlate positively with working senior citizens’ quality of life. The mediating role of hope of success between psychological capital and quality of life was confirmed. The study presented three mechanisms in which hope of success strengthens the relationships between the components of psychological capital and working senior citizens’ quality of life.

**Conclusions:**

The mediating role of hope of success between the remaining components of psychological capital and quality of life confirms pattern posited in the COR theory, namely, that increasing one resource leads to the activation of others, which results in a spiral of personal resources being generated. One the one hand, people who have greater resources have a greater capacity for generating spirals of gains. One the other hand, individuals who lack personal resources are both more exposed to losing them and less capable of starting a spiral of gains in resources.

## Introduction

As a result of the increasing average human life expectancy and the related population growth in many countries, research on factors increasing seniors’ quality of life is becoming particularly important. The quality of life experienced by seniors comprises the following aspects of their existence: physical and psychological (emotional) well-being, the quality of interpersonal relations, personal development, independence, autonomy, social inclusion, individual rights, and material well-being [1]. The analysis of the presented issue falls into the category of *life-span research*, which consists in investigating changes in human behavior across different periods of life, with special attention to critical events that have a decisive impact on the individual’s activity in each of these periods [2]. An event critical for seniors’ functioning is retirement [3]. The reasons for this include the loosening of interpersonal relations, the increased risk of social isolation, and the limitation of activity, which leads to a paradoxical situation when having more free time does not result in maintaining psychomotor fitness and/or social activity [4].

Seniors’ perception of the quality of their life is influenced by diverse subjective factors and by various external circumstances–such as mental attitude towards old age and acceptance of the changes taking place, the outcome and achievements of one’s entire life, the level of energy/vitality, physical fitness, external appearance, psychophysical condition, the severity of health problems (including the experience of chronic illnesses), independent coping with everyday activities, marital status, social and professional activity, engagement and position in the family and in other social groups, social support (including professional support in the form of high-quality medical care), education level, and financial and housing status (financial independence) [5–16].

An interesting issue is the relationships between seniors’ professional activity and their quality of life. It should be noted that the level of professional activity of people in old age varies across European countries. For example, in 2010 its level among Poles aged 50+ was one of the lowest in the European Union [17]. This kind of situation classified Polish society into the category referred to as the culture of “early withdrawal” from the labor market [18]. Analyses also show that the employment rate of older people is systematically growing in Poland. More specifically, in 2002 the employment rate of workers aged 55 to 64 was 26.1% for both sexes, 34.5% for men, and 18.9% for women, while in 2015 the corresponding rates were 44.3%, 54.3%, and 35.5%, respectively. This growth, however, was lower than in other countries of the EU, where the mean value of the employment rate in question in 2015 reached as 53.3% for both sexes jointly, which makes it 10 percent higher than in Poland (the gender-specific rates were 47% for women and more than 60% for men) [19]. In 2017, in the group of people past the retirement age in Poland (60 for women and 65 for men), approximately three-fourths of the population received retirement pension, 13% combined retirement pension with professional career, and 11% worked without receiving retirement or disability pension [20]. The most frequent motivation for continuing to work after retirement was financial. Despite the presence of this type of motivation, a large proportion of seniors (more than 50%) reported that they would like to work even if they were financially independent–for reasons that included satisfaction with work, friendly atmosphere, and good interpersonal relations (both with co-workers and with superiors), employment stability, the meaningfulness of the work they did, the use of their experience and skills, and opportunities to implement their own ideas. At the same time, in the 50+ age group there are people who do not take up professional activity due to poor health condition, insufficient education, the lack of job offers, competence gaps (e.g., related to languages or computer literacy), the lack of certificates confirming adequate skills and/or experience, having no habit of improving their skills all the time, the lack of a developed lifelong learning system, new demands being set for employees to meet due to fast technological changes, and negative stereotypes on the part of employers (e.g., old-age workers as ones who are unable to meet the demands of a specific job, who are more often ill, and who react to changes less flexibly than others) [21].

An important justification for addressing the research problem taken up in this study is the existing results of studies comparing various dimensions of quality of life across sociodemographically and occupationally diverse groups in terms of variables such as social capital or psychological, physical, social, and material well-being. The results of these comparisons showed that seniors’ quality of life was low. In a ranking with 147 possible positions, people aged 60 to 64 ranked 131st, seniors aged above 65 ranked 143rd, and retired senior citizens ranked 135th [22].

The patterns presented above justify looking for relationships between the psychological functioning of working seniors and their quality of life. A concept reflecting the dynamics of seniors’ personality associated with the changes taking place in their life situation is psychological capital (PsyCap). This concept was identified as a factor that is constituted by four personality variables changing because of experiences: self-efficacy, optimism, psychological resilience, and hope of success [23–25]. Psychological capital is a positive psychological state of individual development that is generated by: (1) a belief that one should make careful efforts to succeed in difficult tasks (self-efficacy); (2) a positive attitude regarding the achievement of success at present and/or in the future (optimism); (3) resistance to problems and/or easy/flexible recovery of balance when perceiving the negative outcomes of the difficulties experienced (psychological resilience); (4) perseverance in striving to achieve goals and directing one’s activities to such efforts that increase the likelihood of succeeding (hope of success) [26]. The developmental nature of the personality components of social capital stems from several mechanisms. The first of these is the feeling of certainty that putting effort into the performance of a task will result in success (self-efficacy). The second mechanism stems from a positive attitude to the current and/or future outcomes of the actions undertaken (optimism). The third mechanism is associated with resistance to the stress that accompanies the occurrence of obstacles to and difficulties in actions aimed at the performance of purposeful tasks (psychological resilience). The fourth mechanism manifests itself in consistently striving for the achievement of goals and in looking for alternative paths towards their effective achievement (hope of success) [27, 28]. Thus, the developmental character of the components of psychological capital stems from the fact that, on the one hand, their presence determines “who you are here and now”, and on the other hand it determines “who you can become” in the nearest future.

### General self-efficacy and quality of life

General self-efficacy is the belief in one’s ability to use the cognitive resources, skills, learned solutions, and effort needed to effectively perform a task. People higher in self-efficacy are more likely to treat adversities and/or goals as challenges. Due to the functions mentioned above, self-efficacy is treated as one of the self-regulatory abilities that mediates in setting ambitious goals and ones that are of vital importance in life, making decisions, motivating actions, engaging in behaviors, and controlling one’s activities, which leads to the experience of success in and satisfaction with the tasks performed and to coping with difficult situations constructively. This suggests that the analyzed factor contributes to an increase in quality of life [29–35].

### Dispositional optimism and quality of life

Dispositional optimism is defined as a relatively stable personality tendency (1) to perceive, explain, and evaluate the world and the phenomena taking place in it in positive rather than negative terms and (2) to predict future events (specific) as fortunate rather than unfortunate. Optimists show a positive attitude towards the future even when confronted by negative events, whereas pessimists tend to blame themselves for the negative aspects of their life and to inhibit their actions. These patterns stem from the fact that optimism is marked by a characteristic way of explaining events. It consists in interpreting negative circumstances as external, temporary, and situational and in interpreting positive situations, conversely, as personal, lasting, and universal. As a result of these attributes, a high level of optimism is treated as a personality-based regulatory mechanism in activities aimed at the realization of future goals–among other reasons, this is because it is accompanied by positive emotions, improves physical and psychological well-being, leads to higher engagement in activities aimed at goal achievement, strengthens motivation during the performance of tasks (due to positive outcome expectancy even in situations of mounting adversities), fosters resistance to stressful life events, promotes the use of constructive strategies of coping with stress and, in consequence, helps achieve success and increases quality of life [36, 37].

### Psychological resilience and quality of life

Psychological resilience is a relatively stable personality disposition that contributes to the process of flexibly adapting to the frequently changing demands of life in an appropriate, consistent, and persistent way both by adjusting one’s abilities and skills and by making proper use of the factors present in the environment. The appearance of the signs of positive adaptation also means that the individual in able to cope in a positive way with adversities, conflicts, failures, traumatic events, and/or positive factors associated with progress and growing responsibility. Thus, psychological resilience reflects human capacity to endure even the most negative consequences of stress and comes down to treating difficult situations as challenges to face up to through various forms of engagement. It should also be noted that, according to some theories, a condition for the exemplification of resilience is the presence of increased risk in the individual’s life (e.g., exposure to traumatic events due to family, individual, or community factors). The variable in question is therefore a personality factor that allows individuals to endure stress and/or quickly regain balance after experiencing it. Constructive coping with stress stems from the fact that resilient individuals (i.e., ones with a high level of this variable) are characterized by self-confidence, productivity, independence, sense of humor, patience, determination in action, a high level of inner peace, expression of positive emotions, and openness to new experiences. These attributes contribute to the existence of positive associations between a high level of psychological resilience and satisfaction with life [38–44].

### Hope of success and quality of life

Hope of success reflects self-beliefs according to which (1) the individual will succeed (this gives rise to determination to achieve goals) and (2) this success depends on the ability to use alternative paths for planning and achieving success (e.g., by making goal realization plans or finding ways of overcoming obstacles). What is also characteristic is the simultaneous thinking about goals and about the ways of achieving them–such as seeking alternative options to blocked realization paths that stimulate energy and increase the sense of being in control of events instead of helplessness. The functional link between the above elements thus exists to induce hope of success, which is a positive source of information in difficult circumstances because it results in the situation being evaluated as less threatening; this in turn leads to an increase in motivation to initiate remedial actions [45, 46]. The causative power of hope is exemplified by assertions such as “I can do this” or “I will not stop”. The regulatory functions of hope of success stem, among other things, from the fact that it plays an important role in setting ambitious (though realistic) goals and then striving to accomplish them with determination, positive emotions, and energy to overcome obstacles. The above mechanisms lead to an increase in the effectiveness of behaviors and to constructive adaptation, and thereby to an increase in the likelihood of experiencing high quality of life [47–49].

In the literature on the subject, it is pointed out that the variables making up psychological capital act synergically and generate diverse interactions in different situations and contexts [23]. For this reason, we took up research on the mediating role of hope of success in the relationships between quality of life and the remaining components of psychological capital (self-efficacy, resilience, and optimism). We expected that psychological capital in the form of self-efficacy, resilience, and optimism would have a positive effect on quality of life and that the level of hope of success would increase the likelihood that life difficulties might be successfully resolved thanks to personal abilities, which in turn would translate into better quality of life. Hope of success is an important protective factor for quality of life, which made it reasonable to assume that hope was a way of coping that would enhance quality of life in professionally active seniors. Based on the above assumptions, the research was guided by the following hypotheses as formulated by the researchers:

H1: Psychological capital correlate positively with quality of life in seniors working after retirement.
H2: Hope of success is a mediator between psychological capital and quality of life in seniors working after retirement.
H2a: Hope of success is a mediator between dispositional optimism and quality of life.
H2b: Hope of success is a mediator between resilience and quality of life.
H2c: Hope of success is a mediator between general self-efficacy and quality of life.

## Materials and methods

### Participants and procedure

The study was conducted according to the guidelines of the Declaration of Helsinki, and approved by the Ethics Committee of the Institute of Sociological Sciences of the John Paul II Catholic University of Lublin (protocol code: KEB-IS-2020).

The study included 304 participants of retirement age (60+ in the case of women and 65+ in the case of men). Their mean age was *M* = 65.24 years (*SD* = 9.45). The predominant categories in the sample were men, participants with secondary or elementary education, inhabitants of both towns and cities, and people living in a relationship. Most of the senior citizens worked part-time, predominantly on contracts (order, specific task, or employment contract). Most participants consciously made the decision to continue employment after retirement. Slightly more participants did manual labor than intellectual work (Table 1).

**Table 1:** Participants’ sociodemographic characteristics [N = 304].

Variables		*M*	*SD*
Age		65.24	9.45
		***N***	**%**
Sex	Women	103	33.9
	Men	201	66.1
Education	Higher	97	31.9
	Secondary / elementary	207	68.1
Place of residence	Village	65	21.4
	City / town	239	78.6
Marital status	In a relationship	226	74.3
	Single	78	25.7
Type of work	Blue-collar / manual	176	57.9
	White-collar / intellectual	128	42.1
Employment time	Part-time	190	62.5
	Full-time	114	37.5
Form of employment	Self-employment	26	8.6
	Contract	278	91.4
Plans of employment	Yes	231	76.0
	No	73	24.0

*N* = frequency; % = percentage; *M* = mean; *SD* = standard deviation.

The cross-sectional study conducted in 2020 among seniors working after retirement. Participants were invited to complete a set of questionnaires and then return them personally to research assistants. No time limitations were imposed on the participants. The participants were fully briefed of the aim of study, and their queries were clarified by the researchers. The study was anonymous. Similarly, responses were provided in the presence of trained interviewers. Participants received an explanation and gave their verbal consent to participate in the study. The criteria for inclusion in the sample were: being retired, formal employment (self-employment) on a part-time or full-time basis, intellectual capacity to carry out the study, health status enabling the fulfilment of professional duties (in Poland, there is an obligation of medical examination, verifying positive health status both on taking up a job and during its performance), different levels of education and occupations (in order not to limit the results obtained to one level of education and a specific occupation). As exclusion criteria from the study, the performance of professional duties was treated: 1) based on civil law contracts (mainly commission contracts, called "junk contracts"), because this type of professional contracts—in contrast to formal self-employment and full-time work—is characterised by a short period of work provision, 2) without a formal contract—due to the impossibility of verifying the positive health status of seniors working illegally. The supervisors of the study were the employees of the Institute of Psychology and the Institute of Sociological Sciences of the John Paul II Catholic University of Lublin.

### Variables

#### Quality of life

To assess seniors’ quality of life, we used CASP-19, a questionnaire developed by Higgins, Hyde, Wiggins, and Blane [50]. This measure allows for identifying the areas of human needs that can be seen as domains of quality of life, having both a material dimension and a non-material one. CASP-19 consists of 19 items grouped into four subscales: Control, Autonomy, Self-Realization, and Pleasure. The values of Cronbach’s α for these domains range from .59 to .77. Respondents rate the items on a 4-point scale, from 0 to 3; the maximum total score is 57. Higher scores indicate better quality of life.

#### Self-efficacy

To assess self-efficacy, we used the Generalized Self-Efficacy Scale (GSES) developed by Schwarzer and Jerusalem [51]. According to theory, self-efficacy is commonly understood as being task-specific domain-specific. Some researchers defined self-efficacy as global confidence in one’s coping ability across a wide range of demanding or novel situations. The GSES is a short measure designed to assess the level of individuals’ general belief about their ability to cope with difficult situations and obstacles. It consists of 10 items rated on a 4-point Likert scale, from 1 = *not at all true* to 4 = *exactly true*. The maximum score on this scale is 40. The internal consistency of the GSES was established based on a study including 174 respondents aged 20 to 55; Cronbach’s alpha coefficient was .85.

#### Psychological resilience

To measure resilience, we used the Resilience Assessment Scale (SPP-25) adapted by Ogińska-Bulik and Juczyński [52], which measures the general level of resilience, defined as a personality trait, and the five factors that make it up: (1) perseverance and determination in action, (2) openness to new experiences and sense of humor, (3) personal coping skills and tolerance of negative emotions, (4) tolerance of negative emotions and treating life as a challenge, and (5) optimistic approach to life and focus in difficult situations. These characteristics are assessed on a 5-point scale, from 0 = *definitely not* to 4 = *definitely yes*. In the present study we considered SPP-25 total score, computed as the sum of scores on the five factors, with each factor comprising five items. Total score ranges from 0 to 100; the higher the score, the higher the level of resilience. Cronbach’s alpha was .89.

#### Hope of success

To assess hope of success, we used the Hope of Success Questionnaire (KNS) by Łaguna, Trzebiński and Zięba [53]. Hope of success refers to the strength of the expectation that one’s actions will lead to positive outcomes. It comprises two components: belief in having strong willpower, which consists in the awareness of one’s efficacy, manifesting itself in initiating and persisting in goal realization, and belief in being able to find solutions, which is the awareness of one’s knowledge and intellectual abilities revealed in situations that require inventing or learning new ways of achieving a goal. The questionnaire consists of 12 items; participants rate the degree to which each statement is true about them using a scale from 1 = *definitely not true* to 8 = *definitely true*. This measure has high internal consistency; Cronbach’s α for the whole scale is .82.

#### Dispositional optimism

The Life Orientation Test (LOT-R) in Polish adaptation was used to assess dispositional optimism [54]. LOT-R consists of 10 items; three of them are about optimism (items 1, 4, and 10), three are about pessimism (3, 7, and 9), and four are distractor items (2, 5, 6, and 8), whose scores are not counted. Respondents rate each item by indicating their degree of agreement on a 5-point Likert scale, from 1 = strongly disagree to 5 = strongly agree. Six of the 10 items have assessment value for dispositional optimism. The LOT-R has high internal consistency, with Cronbach’s for the whole scale being .76.

### Statistical methods

We performed a series of correlation analyses and mediation analyses. First, we analyzed zero-order correlations among the variables. The mediation analysis was performed in accordance with the guidelines provided by Preacher and Hayes [55], using IBM SPSS Statistics 26 and PROCESS macro for SPSS. We tested the significance of indirect effects using the bootstrapping procedure. Unstandardized indirect effects were computed for each of the 5,000 bootstrapped samples; the 95% confidence interval was also computed. In accordance with the recommendations provided in the literature, mediation effects were considered significant when the values of the mean estimated indirect effect fell within the 95% confidence interval, with the result that this interval did not include zero. It is assumed that for a mediation effect to be detected there must be significant relations between the independent variable and the mediator (path a) and between the mediator and the dependent variable (path b). In the first step, the aim was to determine the correlation between psychological capital and quality of life (path c). In the second step, the aim was to examine the mediating role of hope of success (path a × b). We used bias-corrected and accelerated 95% confidence intervals. If zero is not included in the 95% CI, the mediating role (c’) is statistically significant. Additionally, classical tests were used to assess the effects of mediation: 1) Sobel test, 2) Aroian test and 3) Goodman test.

Initially, the sample size was expected to be over 250 people [56]. In the end, we surveyed 304 seniors. We calculated whether this sample size would be appropriate. We used the Monte Carlo Power Analysis for Indirect Effects application developed by Schoemann, Boulton and Short [57]. A post hoc Monte Carlo power analysis for the indirect effect (with 1000 replications and 20,000 draws per replication) of psychological capital on quality of life through hope for success shows that we achieved over 80% power for each model.

**Fig 1:**
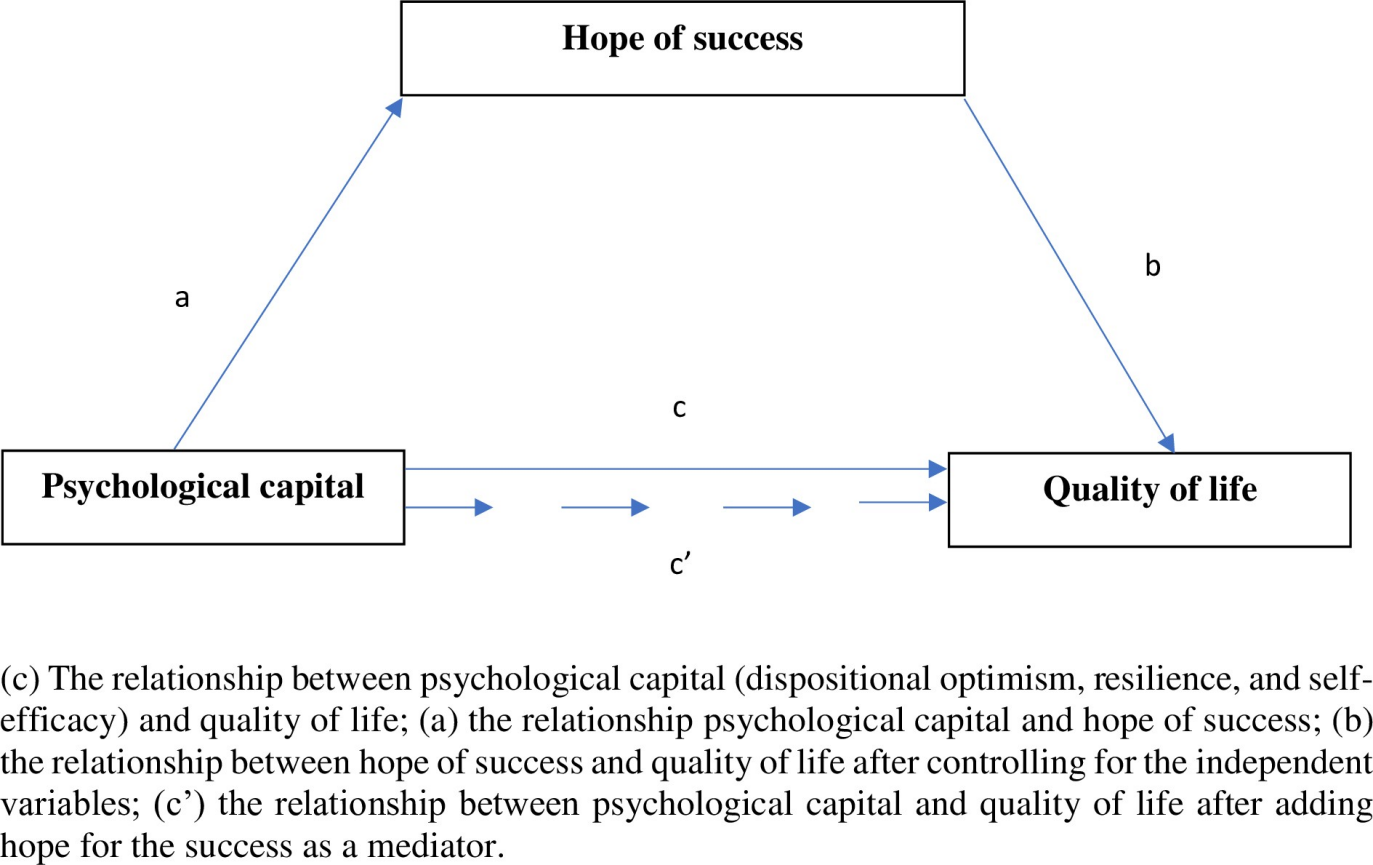
General mediation model.

Fig 1 depicts a simple mediation model with one mediator. Path c represents the total effect of psychological capital (dispositional optimism, resilience, and self-efficacy; three separate models) on quality of life. Path c’ represents both the direct effect of psychological capital on quality of life and the indirect effects of psychological capital on quality of life via hope for the success as a mediator. The specific indirect effect is the product of a and b (path a x path b).

## Results

### Correlations between the analyzed psychological capital and quality of life

We found a moderate positive correlation between total scores on resilience and quality of life (*r* = .40, *p* < .01). Total resilience score was also positively correlated with all CASP-19 domains (C: *r* = .41, *p* < .01; A: *r* = .39, *p* < .01; S: *r* = .44, *p* < .01; P: *r* = .28, *p* < .05). Self-efficacy was positively related to professionally active senior citizens’ general quality of life (*r* = .45, *p* < .01). It correlated positively with three CASP-19 domains, too (C: *r* = .28, *p* < .05); A: *r* = .32, *p* < .01; S: *r* = .41, *p* < .01). We found a positive though weak correlation of dispositional optimism with quality of life (*r* = .18, *p* < .05). Dispositional optimism correlated positively with three CASP-19 domains (C: *r* = .29, *p* < .05); S: *r* = .22, *p* < .05; S: *r* = .22, *p* < .05). Hope of success was moderately and positively correlated with quality of life (*r* = .34, *p* < .01) and with three CASP-19 domains (C: *r* = .32, *p* < .01); A: *r* = .25, *p* < .05; S: *r* = .34, *p* < .01) (Table 2).

**Table 2:** Descriptive statistics and correlations between the analyzed variables (N = 304).

	1	2	3	4	5	6	7	8	9
C [1]	–								
A [2]	.55**	–							
S [3]	.51**	.21*	–						
P [4]	.67**	.39**	.61**	–					
CASP-19 [5]	.77**	.67**	.71**	.75**	–				
Hope for the success [6]	.32**	.25*	.34**	.14	.34**	.56**	–		
Dispositional optimism [7]	.29*	.10	.22*	.22*	.18*	.48**	.33**		
Resilience [11]	.41**	.39**	.44**	.28*	.40**	.55**	.41**		
Self-efficacy [12]	.28*	.32*	.41**	.15	.45**	.51**	.18*	.43**	–
*M* (*SD*)	7.21 (2.99)	10.11 (2.69)	13.16 (2.14)	8.14 (3.66)	38.62 (7.04)	46.72 (8.16)	14.56 (3.80)	70.23 (11.18)	19.60 (5.41)
α	.69	.61	.80	.85	.72	.82	.72	.81	.76

CASP-19 = quality of life; C = Control; A = Autonomy; S = Self-Realization; P = Pleasure;

* p < .05;

** p < .01.

### The mediation role of hope of success

Hypothesis 2 was tested in a series of mediation analyses whose aim was to establish if hope of success was a mediator between psychological capital–in the form of resilience, dispositional optimism, and self-efficacy–and quality of life. In all analyses there was a decrease in the value of beta coefficient in the direct relationship between the analyzed psychological capital and quality of life.

**Fig 2:**
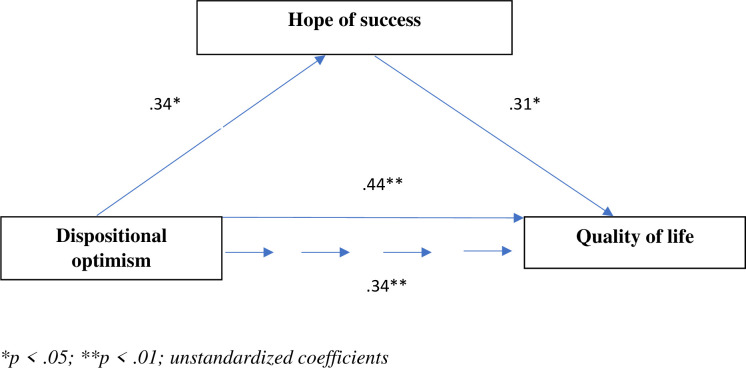
Model of relationships between dispositional optimism, hope of success, and quality of life.

The analyses revealed significant direct associations between dispositional optimism and hope of success (β = .34, *SE* = .05, *p* < .05) and between dispositional optimism and quality of life (β = .44, *SE* = .03, *p* < .01). The direct effect of the mediating variable (hope of success) on quality of life was also significant (β = .31, *SE* = .04, *p* < .05). The direct effect of dispositional optimism on quality of life after adding hope of success as a mediator decreased (β = .34, *SE* = .03, *p* < .01), indicating partial mediation (Fig 2). The results of the bootstrapping analysis confirmed the mediating role of hope of success (BCa 95% CI for a × b of hope of success excluding 0). The mediating role of hope of success confirmed the test results (Sobel test z = 2.259, p = .023; Aroian test z = 2.210, p = .027; Goodman test z = 2.312, p = .021).

We used the following formula to calculate the proportion of its mediating role: a × b / c. The proportion of the mediation effect accounted for by hope of success was 23.9%.

**Fig 3:**
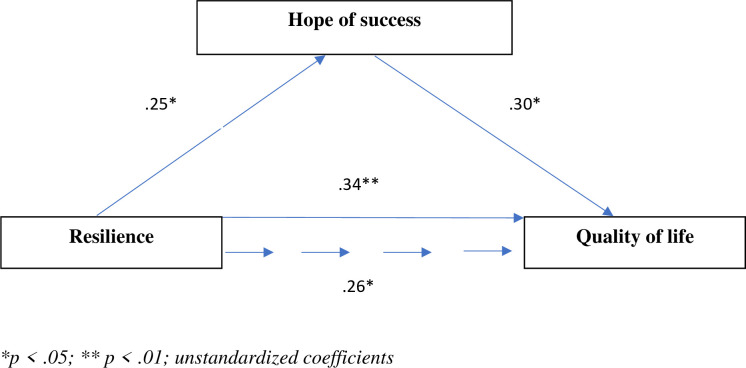
Model of relationships between resilience, hope of success, and quality of life.

Fig 3 shows significant direct associations between resilience and hope of success (β = .25, *SE* = .02, *p* < .05) and between resilience and quality of life (β = .34, *SE* = .05, *p* < .01). The direct effect of the mediating variable (hope of success) on quality of life was also significant (β = .30, *SE* = .03, *p* < .05). The direct effect of resilience on quality of life after adding hope of success as a mediator decreased (β = .26, *SE* = .03, *p* < .05). The results of the bootstrapping analysis and the test results (Sobel test z = 2.525, p = .012; Aroian test z = 2.481, p = .013; Goodman test z = 2.571, p = .010) confirmed the mediating role of hope of success.

The proportion of the mediation effect accounted for by hope of success was 22.1%.

**Fig 4:**
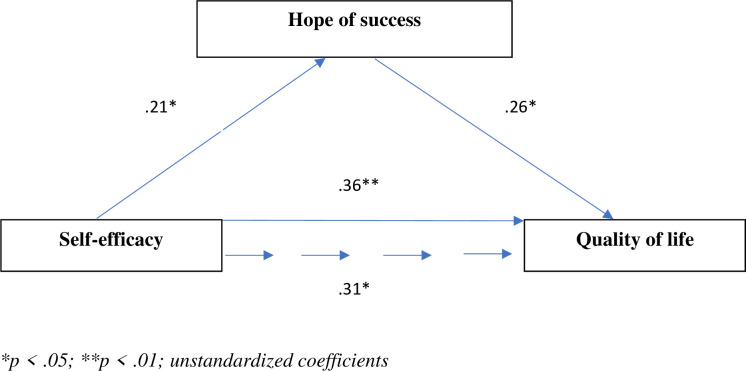
Model of relationships between general self-efficacy, hope of success, and quality of life.

Fig 4 shows significant direct associations between self-efficacy and hope of success (β = .21, *SE* = .06, *p* < .05) and between self-efficacy and quality of life (β = .36, *SE* = .04, *p* < .01). The direct effect of the mediating variable (hope of success) on quality of life was also significant (β = .26, *SE* = .04, *p* < .05). The direct effect of self-efficacy on quality of life after adding hope of success as a mediator decreased (β = .31, *SE* = .04, *p* < .05). The results of the bootstrapping analysis confirmed the mediating role of hope of success. The mediating role of hope of success confirmed the test results (Sobel test z = 4.174, p < .001; Aroian test z = 4.147, p < .001; Goodman test z = 4.202, p < .001).

The proportion of the mediation effect accounted for by hope of success was 15.2%.

## Discussion

Based on the presented results, several conclusions can be formulated. The first one is that the tested components of psychological capital correlate positively with working senior citizens’ quality of life. This means that an increase in the levels of physical and psychological well-being, the quality of interpersonal relations, the experience of personal development, social inclusion, independence/autonomy, perceived individual rights, and material well-being–the dimensions of quality of life in professionally active seniors–co-occurs with the following person-level regulatory mechanisms:

1. strong belief in the possibility of accomplishing the tasks undertaken thanks to one’s personal resources–including abilities, competencies, way of thinking, learned ways of achieving the intended results, and the effort invested in the actions taken (high self-efficacy);
2. perceiving the phenomena taking place in the surrounding world in positive terms, predicting future events as favorable, and explaining negative events as external and temporary while explaining positive ones as personal, lasting, and universal (high dispositional optimism);
3. frequent use of the ability to constructively cope in difficult situations, for example with adversities, conflicts, or failures and with positive factors related to progress and increased responsibility by appropriately adjusting psychological capital and by making use of the factors present in the environment (high psychological resilience);
4. strong belief in the possibility of achieving a goal, high determination in following the goal realization plan, and the ability to find alternative solutions while performing tasks aimed at the achievement of one’s goal in a situation when the existing ways of its realization have been blocked (high hope of success).

The above finding is consistent with the results of other studies, in which it was found that, in people of working age, a high level of psychological capital, comprising the variables mentioned above (self-efficacy, optimism, resilience, and hope of success), had outcomes that included: positive coping with professional and family duties and higher openness to organizational changes [26, 58]; an increase in work efficiency and professional success–resulting, among other things, from an increase in motivation to learn and from using the ability to solve problems in original and creative ways [35, 59–62]; lower employee rotation and higher employability–achieved, for instance, by more frequently preferring adaptive strategies focused on the problem and on active job seeking [63]; an increase in work satisfaction (e.g., thanks to constructive cooperation and the achievement of positive results), organizational engagement, work quality, and good psychological well-being when performing professional duties, and at the same time reducing the symptoms of job burnout, job-related psychological tension/stress, and destructive attitudes and behaviors in the workplace [34, 58, 64, 65].

To sum up the positive associations between high levels of the components of psychological capital and different dimensions of positive attitude to work, it is necessary to stress the finding, reported in the literature, that a high level of psychological capital in professionally active individuals is conducive to the experience of happiness stemming from personal development and from the way they make their professional activities and their whole existence meaningful and valuable (the eudaimonic understanding of happiness) [27, 66, 67].

The results of the present study also support the finding reported in the literature that the variables making up psychological capital can be regarded as resources indispensable to effectively manage one’s own life in the unpredictable social and economic environment, resulting in a high level of quality of life [68–70]. This finding is consistent with the results of research on factors increasing quality of life in seniors. Analyses confirmed that quality of life in this group was high when the following personal factors were present: the predominance of self-efficacy, positive emotions, a high level of motivation to act and achieve important life goals, preference for constructive coping strategies in situations of failure, and emotional stability in problem situations (high resilience). The factors classified as increasing seniors’ quality of life also include the proper functioning of cognitive functions (including a high level of rational appraisal of reality), openness to new experiences (dispositional optimism), and at the same time setting themselves realistic goals to accomplish. The factors listed above increase seniors’ chance of self-fulfillment, self-expression, being useful for others, and making their existence more meaningful [71, 72].

The statistical analyses we conducted support another important finding, which is that hope of success in working seniors performs mediating functions between the remaining components of psychological capital (general self-efficacy, dispositional optimism, and psychological resilience) and quality of life. Based on the statistical patterns found, it is therefore possible to present three mechanisms in which hope of success strengthens the relationships between the components of psychological capital and working senior citizens’ quality of life.

The first mechanism consists in the fact that working seniors’ strong belief in the possibility of achieving a goal, their determination in following the goal realization plan, and their ability to find alternative solutions when the possibilities of achieving the desired outcome have been blocked (high hope of success) strengthen the relationship between high self-efficacy regarding the realization of tasks due to their personal abilities/resources–such as abilities, skills, way of thinking, learned ways of task performance, effort invested in actions (high self-efficacy)–and high levels of physical and psychological well-being, the quality of interpersonal relations, the experience of personal development, the sense of social inclusion, the experience of independence/autonomy, perceived individual rights, and material well-being (high quality of life).

The second mechanism is associated with the fact that working seniors’ strong belief in the possibility of achieving a goal, their determination in following the goal realization plan, and their ability to find alternative solutions when the possibilities of achieving the desired outcome have been blocked (high hope of success) strengthen the relationship of the positive way of perceiving the phenomena taking place in the surrounding world, anticipating favorable future events, and explaining unfavorable events as external and temporary and positive events as personal, lasting, and universal (high optimism) with physical and psychological well-being, the quality of interpersonal relations, experiences suggesting personal development, the sense of social inclusion, the experience of independence/autonomy, perceived individual rights, and material well-being (high quality of life).

The third mechanism refers to the fact that working seniors’ strong belief in the possibility of achieving a goal, their determination in following the goal realization plan, and their ability to find alternative solutions when the possibilities of achieving the desired outcome have been blocked (high hope of success) strengthen the relationship between high capacity for constructive coping in difficult situations by appropriately adjusting personal resources and by making proper use of the factors present in the environment (resilience) and a high level of quality of life in dimensions such as: physical and psychological well-being, the quality of interpersonal relations, the experience of personal development, the sense of social inclusion, the experience of independence/autonomy, perceived individual rights, and material well-being.

The mechanisms presented above are consistent with the assumptions of Hobfoll’s conservation of resources (COR) theory [73]. The basic principle posited in this theory is that a person strives to gain, maintain, support, and protect those resources that are a central value for him or her. At the same time, resources do not exist independently but function in teams/caravans. It can therefore be said that psychological capital is a caravan of resources, made up of self-efficacy, optimism, resilience, and hope of success.

The mediating role of hope of success between the remaining components of psychological capital (self-efficacy, optimism, resilience) and quality of life confirms another pattern posited in the COR theory, namely, that increasing one resource leads to the activation of others, which results in a spiral of resource gains being generated. The way in which psychological capital functions (including the mediating role of hope of success) reflects also other principles based on the COR theory. First, people who have greater resources have a greater capacity for generating spirals of gains. Second, individuals who lack resources are both more exposed to losing them and less capable of starting a spiral of gains in resources [74, 75].

## Limitations

Our study was only exploratory. In further analyses it is worth conducting longitudinal studies, which allow for assessing changes in the dynamics/stability of factors constituting psychological capital in different periods of life in accordance with the methodology of life-span research.

Another aspect of future studies should consist in looking for variables modifying both the components of psychological capital (e.g., social support, work environment resources) and the relationship between psychological capital and quality of life in seniors. Pursuing this direction of research, authors should consider various forms of professional activity–such as volunteer work, receiving old-age pension and at the same time taking up extra work, and continuing to work full-time or part-time despite having reached retirement age. The analysis of relationship between personal capital and the experience of quality of life should focus on those senior citizens who have taken up work for their own pleasure and/or to satisfy the need for self-fulfillment. Further research should consider confounding variables that may influence this relationship, such as education level, place of residence, marital status and health situation. These variables may also be potential moderators of the relationship between psychological capital and quality of life of working seniors.

Another limitation of this study is related to the data collection method. The data collection used self-reported results, which may cause bias. The participants are often biased when they report on their own experiences. Subjects may make the more socially acceptable answer rather than being truthful or may not be able to assess themselves accurately. Nevertheless, when self-reported studies are used correctly, the obtained data can help to elicit a wider range of responses than many other data collection instruments [76]. In future research it is also worth analyzing the components of seniors’ psychological capital in the context of the principles developed based on the conservation of resources (COR) theory–for instance, in terms of gains and losses in psychological capital as a result of various factors and life experiences, in terms of the adjustment of psychological capital to the conditions of the environment (e.g., cultural, professional, communal), or in terms of crossovers of psychological capital with other types of resources. In this kind of research, the mediating and moderating significance of personal capital should be taken into account.
